# Supplementary material for: Differential Expression of Circular RNAs in Rat Brain Regions with Various Degrees of Damage After Ischemia–Reperfusion
Source: Int J Mol Sci. 2025 Oct 30;26(21):10555. doi: 10.3390/ijms262110555 (PMC12608004; doi:10.3390/ijms262110555)
Supplement: Supplementary file 1 [file ijms-26-10555-s001.zip › Supplementary Figure S1.pptx]

## Slide 1
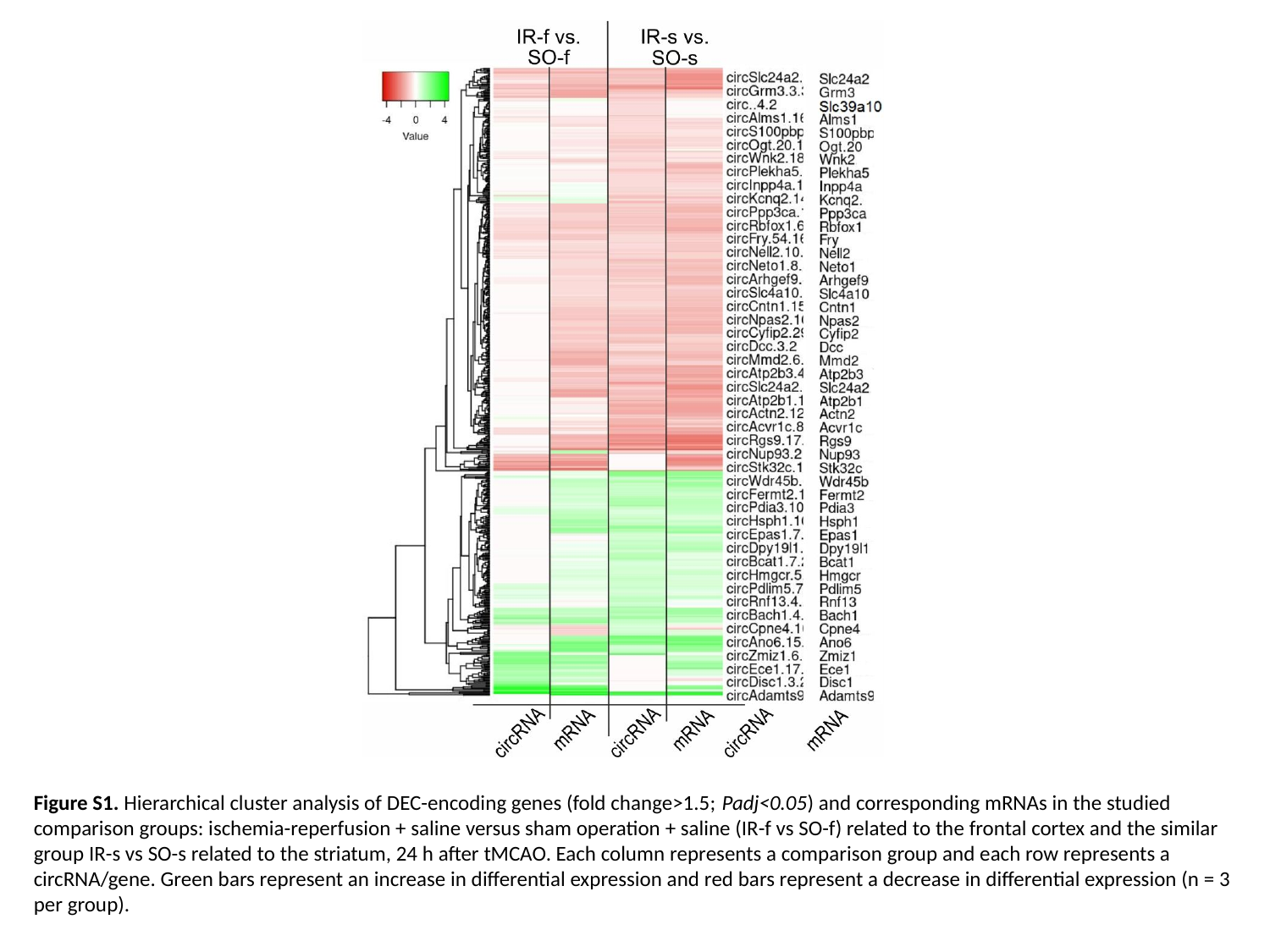

Figure S1. Hierarchical cluster analysis of DEC-encoding genes (fold change>1.5; Padj<0.05) and corresponding mRNAs in the studied comparison groups: ischemia-reperfusion + saline versus sham operation + saline (IR-f vs SO-f) related to the frontal cortex and the similar group IR-s vs SO-s related to the striatum, 24 h after tMCAO. Each column represents a comparison group and each row represents a circRNA/gene. Green bars represent an increase in differential expression and red bars represent a decrease in differential expression (n = 3 per group).
