# Supplementary figures and images for: Differential Expression of Circular RNAs in Rat Brain Regions with Various Degrees of Damage After Ischemia–Reperfusion
Source: Int J Mol Sci. 2025 Oct 30;26(21):10555. doi: 10.3390/ijms262110555 (PMC12608004; doi:10.3390/ijms262110555)

## Slide 1
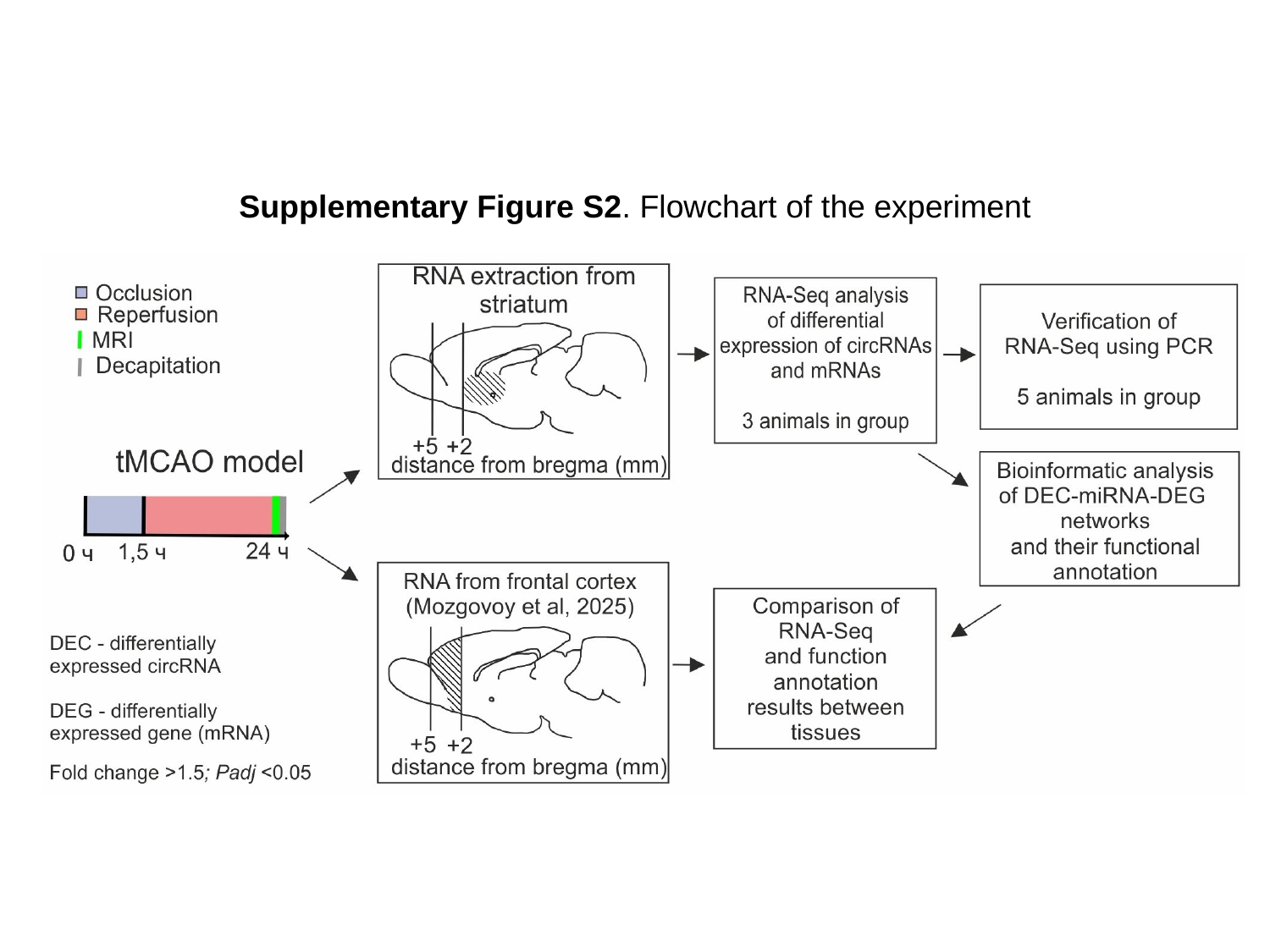

Supplementary Figure S2. Flowchart of the experiment

Supplement: Supplementary file 1 [file ijms-26-10555-s001.zip › Supplementary Figure S2.pptx]
